# Prevalence and factors associated with self-reported kidney disease among Serbian adults: Results of 2013 National Health Survey

**DOI:** 10.1371/journal.pone.0203620

**Published:** 2018-09-12

**Authors:** Dragana Jovic, Nada Dimkovic, Ivana Rakocevic, Katarina Boricic, Dragana Atanasijevic, Milena Vasic

**Affiliations:** 1 Center for Hygiene and Human Ecology, Institute of Public Health of Serbia, Belgrade, Serbia; 2 Clinical Department for Renal Disease, Zvezdara University Medical Center, Belgrade, Serbia; 3 Faculty of Medicine, University of Belgrade, Belgrade, Serbia; 4 Department for Prevention and Control of Non-communicable Diseases, Institute of Public Health of Serbia, Belgrade, Serbia; 5 Center for Health Promotion, Institute of Public Health of Serbia, Belgrade, Serbia; 6 Center for Analysis, Planning and Organization of Health Care, Institute of Public Health of Serbia, Belgrade, Serbia; Postgraduate Medical Institute, INDIA

## Abstract

**Background:**

Data from developing countries on the rates of kidney disease are scarce. The study aimed to estimate population-based prevalence of self-reported kidney disease (SRKD) in Serbia, describe co-occurrence of chronic diseases/conditions/functional limitations in respondents with SRKD and explore association between SRKD and possible risk factors.

**Methods:**

We performed a secondary analysis of 2013 National Health Survey data. Data on a total of 14,587 respondents aged 15 years or older were analyzed using means of descriptive statistics, principal component analysis and logistic regression analysis.

**Results:**

Out of all study respondents, 5.6% (95%CI 5.2–6.0) reported presence of kidney disease. Prevalence of all analyzed morbidities and functional limitations was higher in respondents with SRKD, and they had 8 times higher likelihood of being diagnosed with cirrhosis, 6.3 times higher likelihood of being diagnosed with urinary incontinence, more than 3 times higher likelihood of being diagnosed with degenerative disorder of bone and joint system. For cardiovascular diseases we obtained odds ratios (ORs) from 2.27 (95%CI 2.32–3.44) for heart attack to 2.95 (95%CI 2.43–3.57) for coronary heart disease. Number of co-occurrence patterns of kidney and other chronic diseases/conditions varied depending on inclusion of obesity in models. Logistic regression analysis showed that age explained most part of variability in the prevalence of SRKD and in the prevalence of two morbidities in respondents with SRKD, whereas the presence of three or more morbidities were associated with female gender, aging and low education level.

**Conclusions:**

Our study provided evidence that the presence of kidney disease was significantly associated with socio-demographic, lifestyle characteristics and a number of morbidities in Serbia. There is a need for integrated care and public health interventions, tackling management of NCDs and their risk factors. Detailed well-designed studies, as part of cost-effective preventive approach, are needed for chronic kidney disease screening.

## Introduction

Kidney disease (KD) has become significant contributor to the global burden of morbidity and mortality. Evidence suggest that 8–10% of the world`s adults have some form of kidney damage [1], that the world population prevalence of chronic kidney disease (CKD) is of similar magnitude as of diabetes mellitus (DM) [2–4] and that the prevalence of earlier stages of CKD, as well as the incidence of end-stage KD (ESKD) treated with dialysis, have been increasing during the last two decades worldwide, particularly in the elderly [5–7]. According to the 2010 Global Burden of Disease Study, mortality due to CKD climbed in the list of all causes of deaths worldwide, from 27^th^ position in 1990. to 18^th^ in 2010. [3,8,9]. According to the same source, premature mortality due CKD, with an 82% increase in years of life lost, was the third largest after HIV and AIDS (96%), and DM (93%) [3,8,9].

Reasons for the growth of KD in general and of ESKD in particular, and their association with unfavorable outcomes are complex. Though many KDs are rare and are subject of specialized nephrological care [3], major non-communicable diseases (NCDs) such as DM, hypertension (HTN), cardiovascular diseases (CVD) and obesity are highly prevalent and are most probably reflected on the rise of kidney pathology and ESKD [3,10,11]. This is further complicated by the fact that KD is often referred to as a “silent disease” and may result from lack of early detection and management of other “silent” chronic diseases, including NCDs [11,12]. Accordingly, co-occurrence of kidney and other chronic diseases strongly influence patient`s longer-term morbidity and mortality, quality of life, disease management, burdening patients, the families, provision of healthcare and communities. On the other side, KD *per se* represents risk factor for ESKD and independent risk factor for cardiovascular diseases (CVD), cancer, premature death [12], acute kidney injury (AKI) [13]. Interrelationship between AKI and CKD is also strong: AKI can lead to CKD, and CKD increases the risk of AKI [3,13]. Moreover, science is increasingly recognizing kidney stones, fetal and maternal factors, exposure to nephrotoxins and environmental factors as major threats to kidney health [14], age, gender and race-ethnicity as non-modifiable, and geography, elevated blood pressure and glucose, proteinuria, anaemia, metabolic disturbances, dyslipidaemia, smoking, overweight, obesity as modifiable risk factors for KD. It has been also determined that socio-economic factors (poverty), gender inequality, education, malnutrition, substance abuse, lack of access to primary care all directly increase the risk of KD [12]. Further, a growing association between communicable diseases and NCDs is observed in low and high-income countries [13] and KD is considered to be lying at the interface between communicable and non-communicable diseases [12]. Taking into consideration traditional and nontraditional risk factors for kidney health [14] and synergy between KD and 4 major NCDs [12], the International Society of Nephrology strongly supports the World Health Organization global action plan for the prevention and control of NCDs (2013–2020) and encourages a broad system-wide approach to tackle the problem of KD [12]—surveillance of chronic diseases and their risk factors, implementation of screening programs aimed at early detection and prevention of progression of the disease and implementation of public policies, aimed at developing of prevention strategies for KD [15].

The population of Serbia, out of all health disorders, is burdened most with NCDs and the leading causes of death are almost identical to the causes of death in countries of Europe [16–18]. This is primarily explained by the process of population aging [16,17] and also by characteristics of society in transition, i.e. by socio-economic and environmental determinants of health. Regarding KD, a gradual increase in the number of renal replacement therapy patients was observed in Serbia in the period 2011–2015 (5275 patients were registered in 2011. and 5673 in 2015), and decreasing trend of the number of deceased patients on renal replacement therapy, was observed for the period 2013–2015 (729 deaths were recorded in 2013, 762 in 2014 and 632 in 2015) [18]. According to Serbian Diabetes registry, 21000 people in 2016 addressed to general practitioners in Serbia due to CKD [19]. Regulation on the national Program for prevention, treatment, improvement and control of renal insufficiency and development of dialysis until 2020 was developed in 2011, and National guide of good clinical practice for prevention, diagnosis and treatment of CKD was adopted in 2012. So far, several large-scale studies were carried out assessing the contribution of the prevalence of KD in high-risk population in Serbian healthcare settings [20–22]. However, none of them were population-based, assessing features underlie the prevalence of KD in the general population. The present study elaborates this by assessing population-based prevalence of self-reported KD (SRKD) in Serbia, describing health profile of respondents who reported KD, exploring the distribution of possible risk factors for SRKD using data from latest, 2013 National Health Survey.

## Methods

### Study population and sample

The study utilized data collected in the 2013 National Health Survey—a cross-sectional study that was carried out on a nationally representative sample of the population of Serbia (excluding data on Kosovo and Metohija), and in accordance with the European Health Research Second Wave, World Medical Association Declaration of Helsinki, Serbian regulations on personal data protection and official statistics and Directive 95/46/EC. Details concerning methodology and ethics consideration of the survey are available elsewhere [16,23–27]. In brief, a stratified, two-stage representative sample of the population was selected for the survey, enabling random selection of 10089 households, out of which 6500 were interviewed (household response rate of 64.4%). Of 16474 registered members of the selected households older than 15 years, 14623 were interviewed (response rate of 88.9%). Health information on respondents was obtained by face-to-face interviews, using a standardized questionnaire, as well as anthropometry and blood pressure measurements. Interviews and measurements were carried out in each household by teams consisting of two trained interviewers and a healthcare worker. All respondents signed the informed consent. For children under 18 years of age, consent for participation in the survey was signed by parents. The study protocol was approved by the Review Board of the Ministry of Health of the Republic of Serbia and the Institute of Public Health of Serbia. We analyzed a total of 14587 survey respondents aged 15 years and older.

### Study variables

In order to indentify respondents with kidney disease we used data on self-reported history of KD as measured by the following question: “Have you had kidney problems in the previous 12 months?”. According to the survey protocol [23–26] question on the presence of kidney problems referred to those KDs that fall under the following International Disease Classification codes: N00-N08, N10-N16, N17-N19, N25-N29. All healthcare workers from the interviewer teams were specifically educated to explain respondents to which KD is question related. Participants who answered positively were considered to have KD. We identified 814 respondents with KD and 13773 respondents without KD, and they were analysed for socio-demographic and lifestyle characteristics, and for the presence of other chronic diseases/ conditions and functional limitations. The presence of other chronic diseases/conditions was measured by the following question: “Have you had any of the following diseases or conditions in the previous 12 months?”. Respondents were asked to indicate all diseases/conditions they had. We analyzed data on the presence of bronchial (including allergic) asthma, chronic bronchitis/emphysema (chronic obstructive pulmonary disease, COPD), myocardial infarction or the long term-consequences thereof, coronary heart disease or angina pectoris, hypertension, stroke or the long-term consequences thereof, arthrosis/degenerative joint disease (excluding arthritis), deformity of the lower spine or other chronic problem with the spine, deformity of the neck spine or other chronic problem with the spine, diabetes mellitus, allergy (excluding allergic asthma), cirrhosis, urinary incontinence, depression, malignancy, and hyperlipidemia.

Of the socio-demographic variables we analyzed: gender and age of respondents (categorized into 10 year age groups: 15–24, 25–34 years, etc.); region (identified at the survey level as Vojvodina, Belgrade, Sumadija and Western, Southern, and Eastern Serbia); type of settlement (identified at the survey level as urban or rural); marital status (categorized in two groups-married/living with partner or living without partner), education (categorized as high level-university degree; medium level-three of four years of secondary school; or low level-no education, incomplete primary school, or primary school) and socioeconomic status measured by the household wealth index [28]. According to the calculated wealth index values respondents were classified into five socioeconomic categories or quintiles: poorest, poor, medium, rich and reachest. Of the variables related to lifestyle characteristics of respondents we used smoking status. The smoking status was classified as never-smokers (never smoked), ex-smokers (ceased smoking ⩾1 year earlier) and current smokers. Obesity and functional limitations (frailty, visual impairment, hearing impairment and mobility difficulty) were included in the analysis based on other studies [16, 29]. Body Mass Index (BMI) was calculated from body weight and height measurements (weight in kilograms divided by height in meters squared), and categorized into four categories: underweight (<18.5 kg/m^2^); normal weight (18.5–24.9 kg/m^2^); overweight (25.0–29.9 kg/m^2^) and obese (≥30.0 kg/m^2^). Respondents were defined as „frail” if they had three of the following characteristics: BMI <18.5 kg/m^2^, low level of physical activity (10–29 min per day) and weakness. Weakness was defined using the following survey question: „Do you have difficulties to climb up or go down the 12 steps?”. If the answer was „with some difficulties” or „with much difficulties” or „I am not able”, respondents were classified as weak. Visual impairment was defined as having extreme difficulty when reading text in newspapers or not able to read at all. Hearing impairment was defined as having trouble hearing when having a conversation with another person. Mobility difficulty was defined as experiencing difficulty to walk 500 m [29].

### Statistical analysis

Socio-demographic and lifestyle characteristics of respondents who have/have not reported presence of KD were analyzed using descriptive statistics, the chi-square test, Student t-test, and one-way ANOVA with post-hoc Bonferroni test where appropriate. Categorical variables were described with frequencies and percentages. Prevalence rates of SRKD and of chronic diseases/conditions in respondents in respondents who reported presence of KD were estimated with appropriate 95% confidence intervals (CIs) and weighted using probability sampling weights, calculated to reflect the inhabitants of the Republic of Serbia in 2013. Variance estimates and CIs were used to assess the impact on the precision of stratification and sampling weights using Taylor-series linearization techniques for complex samples. Principal component analysis (PCA) was used to explore the co-occurrence of SRKD and other chronic diseases/conditions, which was also reported by respondents in the survey. Inclusion criteria for the PCA were: prevalence of chronic disease/condition ≥1% in respondents with SRKD; data on chronic diseases/conditions in binary form (1 = presence of the disease and 0 = no disease); application of the principal components method for the extraction of factors by assuming nonparametric distribution of binary data (presence/absence of a disease), the use of scree plots and Monte Carlo simulation (parallel analysis) to determine the optimum number of factors at each step, the Varimax orthogonal rotation method, factor scores > 0.30 as the minimum acceptable value for a correlation that was significant from statistical and clinical standpoints, as well as the identification of at least two diseases per factor. The principal components method was applied to factor extraction, with Eigenvalues >1. Kaiser- Meyer-Olkin was used as a measure of sample adequacy. A previous analysis yielded a proportion of cumulative variance as a measure of the goodness-of-fit model. We analyzed two co-occurrence models: model which included 16 chronic diseases/condition reported by respondents with SRKD [bronchial asthma; chronic bronchitis/emphysema–COPD, myocardial infarction or the long term-consequences thereof, coronary heart disease or angina pectoris, hypertension, stroke or the long-term consequences thereof, arthrosis/degenerative joint disease (excluding arthritis), deformity of the lower spine or other chronic problem with the spine, deformity of the neck spine or other chronic problem with the spine, diabetes mellitus, allergy (excluding allergic asthma), cirrhosis, urinary incontinence, depression, malignancy, and hyperlipidemia], and model which included all previously mentioned chronic diseases and conditions (16 in total), and obesity. Logistic regression analysis was performed to analyze the likelihood of occurrence of chronic diseases/conditions and functional limitation for respondents with and without SRKD, associations between independent variables (age, region, type of settlement, marital status, educational level, wealth index, smoking status, BMI) and the prevalence of SRKD, association between independent variables mentioned above with the number of morbidities (chronic diseases and conditions, 16 in total) in respondents with SRKD. Results are reported with odds ratios (ORs), 95% CIs, and probability (p). All statistical analyses were carried out using SPSS v.20.0 software (SPSS Inc., Chicago, IL, USA) and STATA v.11.1 (StataCorp LP, College Station, TX, USA) with complex sampling design taken into account. Statistical significance was set at a two-sided P value < 0.05.

## Results

In the study sample (14587 respondents aged ≥15 years), 5.6% (95%CI 5.2–6.0) of respondents reported presence of KD. It was reported in all age groups (Table 1). The prevalence of SRKD increased with age, reaching highest values in the 55–64 (22.4%) and 65–74 age group (22.1%). There was a higher proportion of females among respondents with SRKD (61.2%) and a higher proportion of males in respondents without SRKD (51.2%) and the difference was statistically significant. In comparison to respondents without SRKD, participants who reported presence of KD more frequently lived without partner (62.1%) and had middle formal education (45.9%). Regarding BMI, 2.0% of them were underweight, 29,1% of normal weight, 32.7% were overweight and 28.3% were obese. As for the socioeconomic status, one-quarter belong to the poorest quintile (26.4%), and one-fifth to the second (21.3%) and middle (20.2%) quintile. No differences in the type of settlement and according to the region and smoking status were found between subjects with and without SRKD. Two-thirds of respondents who reported presence of KD (75.7%), also reported presence of two or more other chronic diseases. Respondents who reported 2 or more morbidities besides KD, compared to respondents who reported KD only and those who reported 1 other morbidity besides KD, were significantly older, more frequently women, lived without partner, had middle/low formal education and had BMI above normal range. Borderline statistical difference for wealth index (p = 0.05) was found between SRKD respondents having with ≤1 and ≥2 morbidities (Table 1).

**Table 1:** Demographic, socio-economic and lifestyle characteristics of respondents, Republic of Serbia, 2013.

Variables	Self-reported kidney disease		P- value	Self-reported kidney disease and:		P- value
	Yes (n = 814)	No (n = 13773)		≤1 other reported morbidity^§^ (n = 198)	≥2 other reported morbidities^§^ (n = 616)	
	%(95%CI)	%(95%CI)				
**Total**	5.6 (5.2–6.0)	94.4 (94.1–94.8)	0.000	24.3 (21.3–27.2)	75.7 (72.8–78.7)	0.000
**Gender (%)**			0.000			0.004
Men	38.8 (35.4–42.1)	51.2 (50.3–52.0)		47.6 (40.6–54.6)	36.0 (32.2–39.8)	
Women	61.2 (57.9–64.6)	48.8 (48.0–50.0)		52.4 (45.4–59.4)	64.0 (60.2–67.8)	
**Age in categories (%)**			0.000			0.000
15–24	3.1 (1.9–4.3)	14.3 (13.7–14.9)		8.6 (4.6–12.5)	1.3 (0.4–2.3)	
25–34	7.6 (5.8–9.4)	16.4 (15.8–17.0)		20.6 (14.9–26.3)	3.4 (2.0–4.9)	
35–44	8.1 (6.2–9.9)	16.1 (15.5–16.7)		15.5 (10.4–20.5)	5.7 (3.9–7.5)	
45–54	15.5 (13.0–18.0)	16.3 (15.7–17.0)		21.6 (15.9–27.4)	13.5 (10.8–16.2)	
55–64	24.4 (21.4–27.3)	17.9 (17.3–18.5)		16.5 (11.2–21.7)	26.9 (23.4–30.4)	
65–74	22.1 (19.2–24.9)	10.6 (10.1–11.1)		10.2 (5.9–14.4)	25.9 (22.4–29.3)	
75–84	17.2 (14.6–19.8)	7.1 (6.7–7.6)		6.6 (3.2–10.2)	20.6 (17.4–23.8)	
85+	2.1 (1.1–3.1)	1.3 (1.1–14.7)		0.5 (0.0–1.4)	2.6 (1.3–3.9)	
**Region**			0.843			0.392
Vojvodina	27.4 (24.4–30.5)	26.8 (26.0–27.5)		31.2 (24.6–37.8)	26.2 (22.8–29.7)	
Belgrade	22.5 (19.6–25.4)	23.2 (22.5–23.9)		23.6 (17.7–29.6)	22.2 (18.9–25.4)	
Sumadija and Western Serbia	29.0 (25.9–32.2)	28.1 (27.3–28.8)		25.5 (19.4–31.6)	30.2 (26.5–33.7)	
Southern and Eastern Serbia	21.0 (18.2–23.8)	21.9 (21.3–22.6)		19.7(14.1–25.3)	21.5 (18.2–24.7)	
**Type of settlement (%)**			0.772			0.574
Urban	58.9 (55.5–62.3)	59.4 (58.5–60.2)		60.5 (53.7–67.4)	58.3 (54.4–62.2)	
Rural	41.3 (37.7–44.5)	40.6 (39.8–41.5)		39.5 (32.6–46.3)	41.7 (37.8–45.6)	
**Marital status (%)**			0.000			0.000
Married/living with partner	37.9 (34.6–41.3)	39.8 (39.0–40.7)		34.5 (27.9–41.2)	39.0 (35.1–42.9)	
Living without partner ^a^	62.1 (58.7–65.4)	60.2 (59.3–61.0)		65.5 (58.8–72.1)	61.0 (57.1–64.8)	
**Education status (%)**			0.000			0.000
High	13.1 (10.7–15.4)	16.6 (16.0–17.2)		19.0 (13.5–24.5)	11.2 (8.7–13.6)	
Middle	45.9 (42.4–49.3)	54.4 (53.5–55.2)		51.6 (44.5–58.6)	44.1 (40.1–48.0)	
Low	41.1 (37.7–44.4)	29.0 (28.3–29.8)		29.5 (23.1–35.9)	44.8 (40.8–48.7)	
**Wealth index (%)**			0.000			0.005
Reachest	14.0 (11.6–16.4)	19.7 (19.0–20.3)		19.3 (13.7–24.8)	12.3 (9.7–14.9)	
4^th^	18.1 (15.5–20.1)	19.6 (18.9–20.3)		22.7 (16.8–28.6)	16.6 (13.6–19.6)	
Middle	20.2 (17.4–23.0)	19.9 (19.2–20.6)		18.2 (12.7–23.6)	20.9 (17.6–24.1)	
2nd	21.3 (18.5–24.1)	20.7 (20.0–21.3)		14.3 (9.4–19.2)	23.6 (20.2–26.9)	
Poorest	26.4 (23.3–29.4)	20.0 (19.5–20.9)		25.6 (19.4–31.7)	26.6 (23.1–30.1)	
**BMI categories (%)**			0.000			0.000
<18.50 kg/m^2^	2.0 (1.0–3.0)	3.1 (2.8–3.4)		3.7 (1.0–6.3)	1.5 (0.5–2.4)	
18.50–24.99 kg/m^2^	29.1 (26.0–32.2)	39.6 (38.8–40.4)		45.9 (38.9–52.9)	23.7 (20.3–27.1)	
25.00–29.99 kg/m^2^	32.7 (30.0–36.0)	33.9 (33.1–34.7)		28.5 (22.1–34.8)	34.1 (30.1–37.9)	
≥30.00 kg/m^2^	28.3 (25.2–31.4)	19.9 (19.3–20.6)		17.1 (11.8–22.4)	32.0 (28.3–35.7)	
**Smoking status (%)**			0.159			0.027
Non-smoker	40.3 (37.0–43.7)	43.6 (42.7–44.4)		37.9 (31.0–44.7)	41.1 (37.2–45.0)	
Former smoker	15.8 (13.3–18.3)	13.9 (13.0–14.5)		13.2 (8.4–17.9)	16.6 (13.7–19.5)	
Smoker	26.1 (23.1–29.1)	27.5 (26.0–28.2)		33.9 (27.2–40.5)	23.6 (20.2–27.0)	
**Number of morbidities**						
0	10.3 (8.17–12.3)	-	-	-	-	-
1	14.0 (11.6–16.4)	-	-	-	-	-
2	14.5 (12.1–16.9)	-	-	-	-	-
3+	61.2 (57.9–64.6)	-	-	-	-	-

^a^ Unmarried, divorced, or widowed. CI, confidence interval.

^**§**^ analysis included 16 chronic diseases/conditions in total.

Prevalence of all analyzed chronic diseases and functional limitations was significantly higher in respondents who reported presence of KD than those who did not report KD (Table 2). The most prevalent chronic diseases in respondents who reported KD were cardiovascular diseases (76.8%), degenerative disorders of bone and joint system (66.1%) and hyperlipidemia (35.8%). One-fifth of respondents who reported KD (20.9% and 20.1%, respectively) also reported presence of DM and respiratory disease. We found that respiratory disease was 3.7 times, malignancy was 3.6, DM was 3.0 times and urinary incontinence was 9 times more frequently present in SRKD then non-SRKD respondents. Visual impairment (86.9%), mobility difficulty (47.0%) and hearing impairment (30.7%) were the most prevalent functional limitations in SRKD respondents. Prevalence rates of all analyzed chronic diseases were the highest among persons who reported ≥3 morbidities. This was particularly pronounced for HTN, degenerative disorders of bone and joint system and hyperlipidemia (Table 2).

**Table 2:** Prevalence estimates for chronic diseases, conditions and functional limitations in respondents who reported presence of kidney disease, Republic of Serbia, 2013.

Chronic disease/condition/ functional limitation	Respondents with kidney disease ^b^ (n = 730)	Respondents without kidney disease (n = 13773)	P-value^#^	Respondents with kidney disease only (n = 84)	Respondents with kidney disease and 1 other morbidity (n = 114)	Respondents with kidney disease and 2 other morbidities (n = 118)	Respondents with kidney disease and 3 or more other morbidities (n = 498	P-value^##^
	%(95%CI)	%(95%CI)	%(95%CI)	%(95%CI)	%(95%CI)	%(95%CI)	%(95%CI)	
Respiratory diseases^c^	20.1 (17.2–23.0)	5.5 (5.1–5.8)	0.000	-	2.7 (0.3–5.7)	10.9 (5.2–16.6)	26.2 (22.3–30.1)	0.000
Cardiovascular diseases^d^	76.8 (73.7–79.8)	32.2 (31.4–33.0)	0.000	-	43.0 (33.8–52.3)	63.6 (54.8–72.4)	87.6 (84.7–90.5)	0.000
Degenerative disorders of bone and joint system^e^	66.1 (62.6–69.5)	22.7 (22.1–23.4)	0.000	-	24.7 (16.7–32.7)	36.4 (27.6–45.2)	82.6 (79.2–85.9)	0.000
Diabetes mellitus	20.9 (18.0–23.9)	6.9 (6.5–7.3)	0.000	-	3.9 (1.3–7.6)	10.8 (5.0–16.6)	23.3 (23.3–31.2)	0.000
Malignancy	4.7 (3.2–6.3)	1.3 (1.1–1.5)	0.000	-	1.0 (0.8–2.9)	0.7 (0.1–2.3)	6.6 (4.4–8.7)	0.000
Hyperlipidemia	35.8 (32.3–39.4)	12.0 (11.5–12.6)	0.000	-	5.1 (0.9–9.2)	20.1 (12.6–27.5)	46.8 (42.3–51.3)	0.000
Urinary incontinence	26.0 (22.8–29.2)	2.9 (2.6–3.2)	0.000	-	2.3 (0.5–5.1)	15.1 (8.5–21.7)	34.1 (30.0–38.2)	0.000
Frailty	20.7 (17.8–23.7)	6.5 (6.1–6.9)	0.000	7.3 (1.6–13.1)	12.0 (6.0–18.1)	10.6 (4.9–16.2)	25.1 (21.3–28.9)	0.000
Mobility difficulty	47.0 (43.4–50.6)	15.9 (15.3–16.5)	0.000	6.7 (1.3–12.2)	22.9 (15.1–30.7)	28.0 (19.8–36.2)	57.0 (52.8–61.4)	0.000
Visual impairment	86.9 (84.4–89.3)	54.4 (53.6–55.2)	0.000	43.7 (32.8–54.5)	70.9 (62.5–79.4)	85.9 (79.6–92.3)	90.8 (88.2–93.3)	0.000
Hearing impairment	30.7 (27.3–34.0)	11.5 (11.0–12.0)	0.000	5.4 (0.5–10.4)	15.2 (8.5–21.9)	26.9 (18.8–35.0)	35.1 (30.9–39.1)	0.000
Respiratory diseases^c^	%(95%CI)	%(95%CI)	%(95%CI)	%(95%CI)	%(95%CI)	%(95%CI)	%(95%CI)	

^b^ excluding those who reported kidney disease only

^c^ asthma, copd (chronic bronchitis, emphysema)

^d^ hypertension, miocardial infarction, stroke, angina pectoris

^e^ arthrosis, deformity or other chronic problem with the back spine, deformity or other chronic problems with the cervical spine

^#^ statistical difference between respondents with (^b^) and without kidney disease

^##^ statistical difference between morbidity groups

CI, confidence interval.

Factor analysis performed for respondents who reported KD revealed presence of four factors. The first factor was determined by the association between cirrhosis, malignancy, stroke, urinary incontinence and depression. The second factor included deformity or other chronic problems with the neck spine, deformity or other chronic problem with the lower spine, arthrosis-degenerative joint disease and depression. The third included asthma, COPD and allergy and the fourth clustered HTN, coronary heart disease, hyperlipidemia and DM (Table 3).

**Table 3:** Factor scores for the co-occurrence of chronic diseases/conditions in respondents who reported presence of kidney disease, Republic of Serbia, 2013.

Chronic disease/condition	Obesity not included in model				Obesity included in model				
	Factor 1	Factor 2	Factor 3	Factor 4	Factor 1	Factor 2	Factor 3	Factor 4	Factor 5
Cirrhosis	**0.727**		0.296		**0.708**		0.308		
Malignancy	**0.720**				**0.712**				
Stroke	**0.655**				**0.619**				
Urinary incontinence	**0.467**				**0.501**				
Heart attack	**0.450**			0.347	**0.408**			**0.533**	
Deformity or other chronic problems with the neck spine		**0.797**				**0.794**			
Deformity or other chronic problem with the lower spine		**0.792**				**0.791**			
Arthrosis-degenerative joint disease		**0.592**				**0.594**			
Depression	0.334	0.367			0.352	0.370			
Asthma			**0.805**				**0.810**		
COPD			**0.800**				**0.805**		
Allergy			**0.423**				**0.410**		
Hypertension				**0.682**				**0.586**	0.328
Coronary heart disease				**0.633**				**0.763**	
Hyperlipidemia				**0.542**				0.299	**0.421**
Diabetes mellitus				**0.460**	0.317				**0.635**
Obesity	**-**								**0.729**
KMO; p-value	0.776; <0.001				0.772; <0.001				

COPD—chronic bronchitis, emphysema. KMO—Kaiser-Meyer-Olkin measure of sampling adequacy

When obesity was included in the model, factor analysis revealed five factors: factor 1, factor 2 and factor 3 were consisted of the same chronic diseases/conditions; factor 4 combined heart attack, hypertension and coronary heart disease, whereas factor 5 clustered HTN, hyperlipidemia, DM and obesity (Table 3).

Table 4. shows odds ratios (ORs) for associations between chronic diseases, conditions and functional limitations in respondents who reported KD, when controlling for gender, age, education level and smoking status. Respondents who reported KD had 8 times higher likelihood of being diagnosed with cirrhosis, 6.3 times higher likelihood of being diagnosed with urinary incontinence, and more than 3 times higher likelihood of being diagnosed with degenerative disorder of bone and joint system, depression, COPD and deformities or other chronic problems with the lower and neck spine, compared to respondents who did not report KD. For cardiovascular diseases we obtained odds ratios from 2.27 (95%CI 2.32–3.44) for heart attack to 2.95 (95%CI 2.43–3.57) for coronary heart disease. The likelihood of having malignancy, DM, frailty and hearing impairment ranged from 1.5–1.9, respectively (Table 4).

**Table 4:** Odds ratios for associations between chronic diseases, conditions and functional limitations in respondents who reported presence of kidney disease, adjusted for gender, age, education level and smoking status, Republic of Serbia, 2013.

Chronic disease/conditions/functional limitations	Gender (ref. male)	Age (years)	Education (ref. high level)	Smoker (ref. never smoker)	Kidney disease
	OR (95%CI)	OR (95%CI)	OR (95%CI)	OR (95%CI)	OR (95%CI)
Asthma	1.47 (1.24–1.74)**	1.04 (1.03–1.04)**	0.78 (0.61–1.01)	1.42 (1.17–1.71)**	2.46 (1.82–3.23)**
COPD	1.44 (1.27–1.71)**	1.04 (1.03–1.04)**	0.79 (0.61–1.03)	1.35 (1.12–1.64)**	3.13 (2.46–3.99)**
Respiratory disease^c^	1.45 (1.22–1.72)**	1.04 (1.03–1.04)**	0.80 (0.62–1.04)	1.37 (1.14–1.66)**	2.66 (2.13–3.31)**
Heart attack	1.50 (1.21–1.78)**	1.04 (1.03–1.04)**	0.77 (0.59–0.99)*	1.42 (1.18–1.72)**	2.27 (1.63–3.17)**
Coronary heart disease	1.42 (1.20–1.68)**	1.03 (1.02–1.03)**	0.83 (0.64–1.08)	1.43 (1.18–1.73)**	2.95 (2.43–3.57)**
Hypertension	1.39 (1.17–1.65)**	1.03 (1.02–1.03)**	0.81 (0.63–1.05)	1.45 (1.20–1.76)**	2.34 (1.94–2.82)**
Stroke	1.48 (1.25–1.76)**	1.04 (1.03–1.04)**	0.76 (0.59–0.99)*	1.44 (1.19–1.74)**	2.56 (1.79–3.66)**
Cardiovascular disease^d^	1.37 (1.16–1.63)**	1.02 (1.01–1.03)**	0.82 (0.63–1.06)	0.46 (1.21–1.77)**	2.82 (2.32–3.44)**
Arthrosis-degenerative joint disease	1.35 (1.14–1.61)**	1.03 (1.02–1.04)**	0.81 (0.63–1.05)	1.43 (1.18–1.73)**	2.40 (1.97–2.93)**
Deformity or other chronic problems with the neck spine	1.28 (1.08–1.52)*	1.03 (1.02–1.04)**	0.80 (0.61–1.03)	1.37(1.13–1.66)**	3.01 (2.52–3.60)**
Deformity or other chronic problem with the lower spine	1.32 (1.11–1.57)**	1.03 (1.02–1.04)**	0.83 (0.64–1.07)	1.38 (1.14–1.67)**	3.05 (2.58–3.61)**
Degenerative disorder of bone and joint system^e^	1.25 (1.05–1.48)*	1.03 (1.02–1.04)**	0.83 (0.64–1.07)	1.34 (1.10–1.62)*	3.41 (2.87–4.05)**
Diabetes mellitus	1.49 (1.26–1.77)**	1.04 (1.03–1.04)**	0.78 (0.60–1.01)	1.42 (1.17–1.71)**	1.88 (1.51–2.33)**
Allergy	1.37 (1.15–1.63)**	1.04 (1.03–1.04)**	0.74 (0.57–0.95)*	1.41 (1.16–1.71)**	2.91 (2.38–3.57)**
Cirrhosis	1.49 (1.26–1.77)**	1.04 (1.03–1.04)**	0.78 (0.60–1.00)*	1.42 (1.18–1.72)**	8.11 (4.01–16.39)**
Urinary incontinence	1.56 (1.31–1.85)**	1.03 (1.02–1.04)**	0.82 (0.63–1.07)	1.42 (1.17–1.72)**	6.31 (5.02–7.92)**
Depression	1.36 (1.15–1.61)**	1.03 (1.02–1.04)**	0.81 (0.62–1.05)	1.35 (1.11–1.63)*	3.31 (2.68–4.09)**
Malignancy	1.46 (1.23–1.73)**	1.03 (1.02–1.04)**	0.75 (0.58–0.97)*	1.42 (1.18–1.72)**	1.96 (1.25–3.05)*
Hyperlipidaemia	1.39 (1.17–1.65)**	1.03 (1.02–1.04)**	0.76 (0.58–0.98)*	1.39 (1.15–1.69)**	2.40 (2.01–2.88)**
Frailty	1.43 (1.20–1.69)**	1.04 (1.03–1.04)**	0.80 (0.62–1.03)	1.42 (1.17–1.72)**	1.77 (1.41–2.21)**
Mobility difficulty	1.39 (1.18–1.65)**	1.03 (1.02–1.04)**	0.87 (0.67–1.13)	1.43 (1.18–1.73)**	2.03 (1.67–2.48)**
Visual impairment	1.39 (1.17–1.65)**	1.03 (1.02–1.03)**	0.70 (0.55–0.91)	1.35 (1.12–1.63)*	2.12 (1.67–2.69)**
Hearing impairment	1.51 (1.27–1.78)**	1.03 (1.03–1.04)**	0.79 (0.61–1.02)	1.44 (1.19–1.74)**	1.54 (1.25–1.89)**

COPD-chronic bronchitis, emphysema

^c^ asthma, copd (chronic bronchitis, emphysema)

^d^ hypertension, miocardial infarction, stroke, angina pectoris

^e^ arthrosis, deformity or other chronic problem with the back spine, deformity or other chronic problems with the cervical spine; OR, Odds Ratio; CI, confidence interval; Ref, reference category.

*p<0.05

** p<0.01.

Estimated changes in odds ratios (ORs) for association between socio-demographic and lifestyle variables, and the prevalence of SRKD in univariate model (model 1) and various multivariate models (model 2 –which included gender, age, region, type of settlement and marital status; model 3 –which included gender, age, education status and wealth index; model 4 –which included gender, age, smoking and nourishment status; model 5 –which included all interactions of socio-economic and lifestyle variables, irrespective of their significance shown in univariate model) are shown in Table 5. According to univariate logistic regression analysis, the independent effect of age explains the most part of variability in the prevalence of SRKD, but this variability may be also explained by the independent effects of gender, marital status (living without partner), education, socio-economic status measured by wealth index and by increased body weight (both overweight and obesity). All multivariate logistic regression analyses confirmed significant association between the reported presence of KD and gender and age. Although smoking status did not show association with SRKD in univariate model, it appeared as significantly associated with SRKD, in model 4 and in the last generated model, together with obesity. There are other evidences on significant interactions from the last generated model. Middle-aged and old aged participants in comparison to youngest, females towards men, current smokers compared to non-smokers and obese compared to normal weighted participants were significantly more likely to report KD.

**Table 5:** Unadjusted and adjusted odds ratios (ORs) for associations between socio-demographic, lifestyle variables and the prevalence of self-reported kidney disease, Republic of Serbia, 2013.

Variables	Model 1	Model 2	Model 3	Model 4	Model 5
	OR (95%CI)	OR (95%CI)	OR (95%CI)	OR (95%CI)	OR (95%CI)
**Gender**					
Men	Ref.	Ref.	Ref.	Ref.	Ref.
Women	1.51 (1.30–1.74)**	1.39 (1.20–1.61)**	1.37 (1.23–1.75)**	1.47 (1.24–1.75)**	1.42 (1.19–1.1.69)**
**Age (in categories)**					
15–24	Ref.	Ref.	Ref.	Ref.	Ref.
25–34	2.14 (1.34–3.41)**	2.09 (1.29–3.38)**	2.28 (1.08–3.20)**	1.86 (1.08–3.19)**	2.02 (1.15–3.53)**
35–44	2.31 (1.46–3.67)**	2.23 (1.37–3.65)**	2.42 (1.32–3.82)**	2.25 (1.32–3.82)**	2.41 (1.38–4.24)**
45–54	4.36 (2.83–6.71)**	4.19 (2.63–6.68)**	4.51 (2.22–6.12)**	3.69 (2.22–6.12)**	3.93 (2.28–6.78)**
55–64	6.27 (4.13–9.53)**	6.01 (3.80–9.51)**	6.34 (3.39–9.07)**	5.54 (3.39–9.07)**	5.73 (3.35–9.80)**
65–74	9.61 (6.30–14.65)**	9.14 (5.73–14.56)**	9.38 (5.06–13.68)**	8.32 (5.06–13.69)**	8.34 (4.83–14.40)**
75–84	11.12 (7.24–17.11)**	10.46 (6.50–16.83)**	10.51 (5.64–15.71)**	9.42 (5.64–15.71)**	9.13 (5.20–16.04)**
85+	7.50 (3.98–14.14)**	6.91 (3.55–13.45)**	6.92 (3.41–16.87)**	7.58 (3.41–16.87)**	7.37 (3.19–17.01)**
**Region**					
Vojvodina	1.06 (0.86–1.29)	1.07 (0.87–1.32)	-	-	0.95 (0.74–1.21)
Belgrade	Ref.	Ref.	-	-	Ref.
Sumadija and Western Serbia	1.07 (0.87–1.30)	1.06 (0.86–1.31)	-	-	0.96 (0.75–1.23)
Southern and Eastern Serbia	0.99 (0.80–1.22)	0.95 (0.76–1.19)	-	-	0.90 (0.75–1.16)
**Type of settlement**					
Urban	Ref.	Ref.	-	-	Ref.
Rural	1.02 (0.88–1.18)	0.94 (0.80–1.09)	-	-	0.85 (0.69–1.05)
**Marital status**					
Married/living with partner	Ref.	Ref.	-	-	Ref.
Living without partner	2.50 (2.04–3.05)**	1.04 (0.83–1.32)	-	-	0.99 (0.76–1.29)
**Education status**					
High	Ref.	-	Ref.	-	Ref.
Middle	0.60 (0.51–0.70)**	-	0.91 (0.76–1.09)	-	0.84 (0.68–1.03)
Low	0.56 (0.46–0.70)**	-	0.78 (0.59–1.00)*	-	0.73 (0.54–0.98)*
**Wealth index**					
Reachest	Ref.	-	Ref.	-	Ref.
4^th^	1.30 (1.01–1.67)	-	1.11 (0.86–1.44)	-	1.16 (0.82–1.64)
Middle	1.43 (1.12–1.82)*	-	1.13 (0.88–1.46)	-	1.09 (0.79–1.48)
2nd	1.45 (1.14–1.85)*	-	1.05 (0.81–1.37)	-	1.11 (0.82–1.50)
Poorest	1.84 (1.45–2.32)**	-	1.10 (0.84–1.44)	-	1.09 (0.81–1.45)
**Smoking status**					
Non-smoker	Ref.	-	-	Ref.	Ref.
Former smoker	1.26 (0.99–1.51)	-	-	1.24 (0.99–1.55)	1.25 (1.0–1.58)
Smoker	1.03 (0.86–1.23)	-	-	1.39 (1.14–1.70)**	1.38 (1.13–1.69)**
**BMI categories**					
<18.50 kg/m^2^	0.87 (0.52–1.46)	-	-	0.87 (0.45–1.68)	0.86 (0.45–1.66)
18.50–24.99 kg/m^2^	Ref.	-	-	Ref.	Ref.
25.00–29.99 kg/m^2^	1.32 (1.10–1.57)**	-	-	1.10 (0.90–1.35)	1.10 (0.89–1.35)
≥30.00 kg/m^2^	1.93 (1.60–2.33)**	-	-	1.35 (1.09–1.68)**	1.33 (1.07–1.65)*

Model 1: Univariate association.

Model 2: Multivariate model for gender, age, region, type of settlement and marital status.

Model 3: Multivariate model for included gender, age, education status and wealth index.

Model 4: Multivariate model for included gender, age, body mass index and smoking status.

Model 5: In this multivariate model we included all socio-demographic and lifestyle variables.

Ref, reference category; OR, Odds Ratio

* p<0.05

** p<0.01.

In Table 6. univariate and multivariate logistic regression models for associations between socio-demographic, lifestyle variables and the number of chronic morbidities in respondents with SRKD are presented. According to logistic regression analysis, the independent effect of age explained the most part of variability in the prevalence of two reported KD co-morbidities, whereas the prevalence of three or more reported KD co-morbidities were associated with female gender, aging, region, low level of education and obesity.

**Table 6:** Unadjusted and adjusted odds ratios for associations between socio-demographic, lifestyle variables and the number of morbidities in respondents who reported presence of kidney disease, Republic of Serbia, 2013.

Variables	2 vs. none or one morbidity^#^		3 or more vs. none or one morbidity^#^	
	Univariate association	Multivariate association	Univariate association	Multivariate association
	OR (95%CI)	OR (95%CI)	OR (95%CI)	OR (95%CI)
**Gender**				
Men	Ref.	Ref.	Ref.	Ref.
Women	1.11 (0.70–1.75)	1.66 (0.88–3.14)	1.78 (1.27–2.49)**	1.81 (1.13–2.88)*
**Age (years)**	1.04 (1.02–1.06)**	1.05 (1.02–1.07)**	1.07 (1.06–1.09)**	1.07 (1.05–1.09)**
**Region**				
Vojvodina	0.68 (0.35–1.30)	0.30 (0.12–0.73)	0.96 (0.61–1.51)	0.51 (0.26–0.98)*
Belgrade	Ref.	Ref.	Ref.	Ref.
Sumadija and Western Serbia	1.05 (0.56–2.00)	0.76 (0.33–1.77)	1.32 (0.82–2.10)	0.82 (0.42–1.60)
Southern and Eastern Serbia	1.29 (0.67–2.50)	0.86 (0.38–1.97)	1.13 (0.68–1.86)	0.79 (0.39–1.56)
**Type of settlement**				
Urban	Ref.	Ref.	Ref.	Ref.
Rural	0.95 (0.59–1.51)	1.21 (0.57–2.56)	1.13 (0.81–1.59)	1.03 (0.60–1.76)
**Marital status**				
Married/living with partner	Ref.	Ref.	Ref.	Ref.
Living without partner	1.56 (0.86–2.84)	0.61 (0.27–1.40)	2.54 (1.63–3.95)**	0.80 (0.41–1.52)
**Education status**				
High	Ref.	Ref.	Ref.	Ref.
Middle	0.99 (0.59–1.65)	2.03 (0.86–4.76)	0.50 (0.34–0.72)**	0.68 (0.39–1.18)
Low	0.72 (0.36–1.46)	1.30 (0.43–3.97)	0.34 (0.20–0.56)**	0.49 (0.23–1.06)*
**Wealth index**				
Reachest	Ref.	Ref.	Ref.	Ref.
4^th^	0.86 (0.42–1.77)	1.40 (0.43–4.42)	1.95 (1.15–1.3.30)*	1.07 (0.43–2.66)
Middle	0.89 (0.42–1.90)	1.10 (0.38–3.21)	3.05 (1.70–5.46)**	1.74 (0.74–4.08)
2nd	0.52 (0.72–3.20)	0.83 (0.30–2.30)	2.20 (1.25–3.85)	1.45 (0.67–3.13)
Poorest	0.91 (0.45–1.83)	0.60 (0.23–1.54)	1.28 (0.74–2.23)	0.91 (0.43–1.95)
**Smoking status**				
Non-smoker	Ref.	Ref.	Ref.	Ref.
Former smoker	1.32 (0.67–2.62)	1.06 (0.48–2.42)	1.13 (0.67–1.88)	0.59 (0.84–2.99)
Smoker	0.63 (0.35–1.13)	0.95 (0.47–1.90)	0.65 (0.43–0.96)*	1.32 (0.79–2.20)
**BMI categories**				
<18.50 kg/m^2^	1.06 (0.27–4.18)	1.24 (0.14–11.10)	0.66 (0.21–2.04)	0.55 (0.11–2.86)
18.50–24.99 kg/m^2^	Ref.	Ref.	Ref.	Ref.
25.00–29.99 kg/m^2^	1.52 (0.86–2.69)	1.33 (0.65–2.71)	2.59 (1.72–3.90)**	2.02 (1.20–3.42)**
≥30.00 kg/m^2^	2.74 (1.50–4.99)**	3.70 (1.71–8.04)**	3.92 (2.45–6.24)**	2.94 (1.61–5.36)**

# Excluding functional limitations.

Multivariate models included all socio-demographic and lifestyle variables irrespective of their significance in univariate model.

OR, Odds Ratio; CI, confidence interval; Ref, reference category.

*p<0.05

** p<0.01.

## Discussion

According to our knowledge this is the first study that describes profile of adults with SRKD in the general population in Serbia. Its main strengths are the large representative sample of the population, from which our study respondents originate, and statistical method used. The proportion of respondents who reported presence of KD in our study is 5.6% and is smaller than in screening studies previously carried out in Serbia [19–22]. If we accept that around 10% of worldwide population have some degree of kidney damage, than it may mean that around 4.4% of our population is undiagnosed. It is well known that KD in its earliest stages is usually asymptomatic, that it is often overlooked and that most patients, even in at risk populations, are unaware of the presence of KD. Thus, our population-based study supports these observations and highlights the importance of screening procedures aimed at detecting KD in its earliest stage.

Our logistic regression models confirmed older age, female gender and smoking status as independent predictors for the presence of KD. Subjects older than 60 years without HTN or DM are proposed as a target population by KDOQI guidelines [23]. However, physiologic age-related decline in glomerular filatration rate (GFR) and underestimation of GFR by equations based on serum creatinine in healthy older patients are the most common reasons for over-diagnosis of KD, particularly for CKD in the elderly. Reduction in mean creatinine clearance, despite no difference in serum creatinine, can be explained by the so-called “senile sarcopenia” and by reduced protein intake. Therefore, current limit of KD (60 ml/min/1.73 m^2^) [30] may not be necessarily true for a group of elderly. Still, reduced kidney function along with a normal aging process has an important role in daily clinical practice in regard to drug dosing, kidney donor selection, and the definition of the risk of AKI due to a reduced renal reserve. Hence, our results indicate that elderly certainly need to remain an important group for KD screening.

Another finding of our study is that obesity may be associated with higher risk of incident KD. Recent developments in pathophysiology of obesity-related KD indicated that chronic inflammation and abnormal lipid metabolism contribute to kidney cell injury. As this risk extends to those who are metabolically healthy, obesity also contributes to the risk for KD, independently of the metabolic syndrome. Large cohort studies suggested that obesity (BMI≥ 30 kg/m^2^), especially in the context of metabolic syndrome and insulin resistance, is associated with higher risk of *de novo* KD [31]. In a national cohort of more than 3 million US veterans without previously known renal insufficiency [eGFR >60 ml/min/1.73 m^2^), BMI higher then 30kg/m^2^ was associated with loss of kidney function across different ages [32]. Thus, our results suggest that paying attention on screening for KD in obese individuals is of great importance, as obesity remains a significant risk factor for KD.

During the last 5 years several screening studies in Serbia were carried out in order to detect KD in patients who were at risk for KD (those with HTN, DM, and in patients older than 60 years without HTN and DM) [20–22], using questionnaire, blood pressure measurement, single dipstick measurement for microlabuminuria (MAU) and proteinuria, and GFR estimated by Modification of Diet in Renal Disease (MDRD) Study equation. The study of Djukanovic et al. [21] comprising 1617 patients without previously known KD, who came for regular check-ups to their general practitioners in 13 Belgrade primary health centers, showed that among the respondents, 26% had MAU, 10% had proteinuria and 23% had eGFR less than 60 mL/min. Despite different study design, multivariate logistic regression analysis of that study revealed findings similar to our study—female gender, age, smoking status and duration of HTN were associated with the lower eGFR [20]. Our results are also in agreement with the results of National Health and Nutrition Examination Survey (NHANES), where CKD among U.S. individuals with no ESRD was more frequent among women, the proportion of individuals with albuminuria was substantially higher in women (10.2%) than men (8.6%), and the proportion of patients with eGFR *<*60 ml/min per 1.73 m2 was higher in women (7.7%) than in men (5.6%) [33]. Our study also confirmed that female gender is independent predictor of KD.

Important point of our study is potential association between co-morbid conditions and the prevalence of SRKD. Although different guidelines proposed diverse at risk populations for screening, patients with HTN or DM are generally accepted as relevant ones [34–36]. Indeed, previous Serbian studies have shown that patients with HTN, DM and older than 60 years had high prevalence of MAU/proteinuria (30%, 64% and 54%) and decreased eGFR (25%, 12% and 21%). However, data about association of CKD and other diseases are less clear. Our analysis confirmed high association of KD with chronic diseases and clinical conditions in the logistic regression analysis. CVD may be associated with an increased incidence of acute and chronic renal diseases as a consequence of cardio-renal syndrome type 1 and 2 [37].

Present study confirmed that KD is highly associated with CVD, HTN, coronary artery disease, heart attack and stroke (OR ranged from 2.27–2.95). Hyperlipidemia is not only CVD risk factor, but also risk factor for CKD [38], so the results of our research are not surprising. As far as DM is concerned, our results show that it almost doubles the risk for KD (OR 1.88, 95%CI 1.51–2.33). ORs values for the association between KD and CVD, and with DM in our study are smaller than those obtained by Luxembourg study (ORISCAV-LUX) [39], where HTN and DM were associated with more than 3-fold and 4-fold higher risks of KD (AOR 3.46,95%CI 1.92–6.24, P < 0.001) and (AOR 4.45,95%CI 2.18–9.07, P < 0.001), respectively. Again, our study had different design and was not based on screening parameters (MAO, eGFR). However, the prevalence of SRKD in our study was almost the same in the 45–54 and 75–84 year group (15.5% and 17.2%, respectively) and highest in the 55–64 and 65–74 year group (24.4% and 22.1%, respectively), which is occurring in those age groups where the prevalence of HTN and DM is highest in Serbia [16].

Another important finding of the present study is association of KD with broad spectrum of chronic diseases, apart from HTN and DM. The highest risk factor for KD, which is also the highest risk for CKD, was obtained for cirrhosis (OR 8.1, 95%CI 4.01–16.39) and urinary incontinence (OR 6.31, 95%CI 5.02–7.92). Also, some other diseases tripled the risk for KD (depression, COPD and deformity/degenerative disorders of spine and joint system) while asthma, allergy and hyperlipidaemia doubled the risk for KD. In addition, patients with functional limitations were also at risk to develop KD. KD is a disorder frequently suffered by cirrhotic patients. In the clinical context of the patients with liver cirrhosis, accurate evaluation of the renal function is potentially crucial since it can lead to early diagnosis of both acute kidney injury and chronic KD and to reliable characterization of the renal status of the patient before performing a liver transplantation [40]. Also, urinary incontinence which may be a consequence of the broad spectrum of conditions, may when left untreated, has a potential for causing deterioration of renal function over time [41]. Connection between KD and depression is well known and prevalence of depression and suicidal ideation increases proportionally with renal function decline, beginning from early stages of chronic KD [42]. However, this study is not designed to show the exact relationship between the two diseases or their cause-and-effect relationship which could have an impact on the screening strategy. Therefore, more data on this topic have to be targeted in the future.

It is well known that reduced lung function is associated with clinical outcomes such as cardiovascular disease. However, little is known about its association with incident KD and ESKD.Recent results from Atherosclerosis Risk in Communities (ARIC) Study that included 14,946 participants aged 45 to 64 years at baseline that were followed for median of 23.6 years revealed that reduced lung function, particularly lower percent-predicted forced vital capacity, is independently associated with KD progression. Findings suggested a potential influence of reduced lung function to the development of progressive KD and a need for monitoring kidney function in persons with reduced lung function [43]. While data about association between KD and allergy are not know, some reports confirm that systemic inflammation in asthma plays a significant role in the development of other diseases. By analyzing 2,354 subjects with persistent asthma during six-year period, authors confirmed that 9.6% developed proteinuria and 3.1% progressed to eGFR <60 ml/min/1.73 m^2^. Therefore, it is advisable to check kidney function in patients with long-lasting and poorly controlled asthma.

Frailty is a condition usually found in elderly people, and its prevalence among community-dwelling people increases steadily with age, being 4–7% in old people, and 9–26% in very old people. Moreover, female gender and chronic diseases also increase the frailty phenotype prevalence [44]. Pathophysiology of frailty include oxidative stress, chronic inflammation, immune activation, as well as musculoskeletal and endocrine alterations [45]—well known factors of KD progression. Coexistence of CKD and frailty has been shown to further increase risks of falls, fractures, hospitalization, and mortality [44–46]. Given the complicated interaction between frailty and KD, it is difficult to determine the causal relationship between these two conditions, but it is certain that together they make a *circle vicious* with negative outcome of the patients. Therefore, the detection or prevention of fragility is an important step to avoid or prevent KD especially in the elderly population. Our results clearly link these two clinical conditions.

According to our results, degenerative bone and joint disorders obviously bear a high risk of coexistence with KD, since they encounter approximately 66% of patients with KD. The questionnaire certainly could not distinguish between the etiology of these disorders. Many of these patients often use potentially toxic non-steroidal anti-inflammatory drugs due to subjective symptoms. Finally, this group of diseases is the most common in elderly, and the question arises as to whether the association with the KD is causative or it is epiphomenon.

Several limitations of this study need to be observed. First, our study was based on cross-sectional survey, not allowing to overview the causal links between the analyzed variables. Next limitation is related to measurement of bias, because presence of kidney and other chronic diseases were reported by the respondents. In this study respondents were reporting presence of KD, not medical diagnosis of KD. Thus, we could not differentiate types of KD from which respondents were suffering from. With assumption that there is an association between recent acute illness/ exacerbation of the existing disease and reporting on a disease, and that sicker people may be more likely to seek medical help and learn more about their illnesses [16], prevalence of reporting on chronic diseases other than the kidney might be affected in the survey. Lastly, the number of examined chronic diseases/conditions is relatively small. Our study included 16 available due to the survey protocol, unlike others, which due to availability of EDCs System, could involve significantly more diseases in the analysis. Nevertheless, our findings expand the current knowledge on KD in the general population in Serbia.

## Conclusions

The data presented provided evidence that the presence of kidney disease was significantly associated with socio-demographic, lifestyle characteristics and a number of morbidities in Serbia. In the light of synergy between NCDs and KD, their risk factors and complications which involve most organ systems but can be prevented, awareness on the importance of coping actions for KD and the use of accurate methods for enabling timely diagnosis of KD, are needed. Detailed well-designed studies, as part of cost-effective preventive approach, can open a new horizon for CKD screening.
